# Oral Health Conditions and Quality of Life Among Schoolchildren in Rural Tanzania: A Cross-Sectional Study

**DOI:** 10.3390/children13040525

**Published:** 2026-04-09

**Authors:** Kyra Michels, Sebastian Hinz, Anders Henningsen, Simon Megiroo, Werner Kronenberg, Wolfgang Bömicke, Rita Bensel, Tobias Bensel

**Affiliations:** 1 Department of Reconstructive Dentistry and Gerodontology, School of Dental Medicine, University of Bern, Freiburgstrasse 7, 3010 Bern, Switzerland; kyra.michels@unibe.ch (K.M.); tobias.bensel@unibe.ch (T.B.); 2 Division of Regenerative Orofacial Medicine, Department of Oral and Maxillofacial Surgery, University Hospital Hamburg-Eppendorf, Martinistraße 58, 20251 Hamburg, Germany; henningsen@elbe-mkg.de; 3 Health Department, ELCT/NORTH CENTRAL DIOCESE, Arusha P.O. Box 16173, Tanzania; s2003megiroo@yahoo.co.uk; 4 Ilembula Lutheran Hospital, Ilembula P.O. Box 14, Tanzania; werner-kronenberg@web.de; 5 Department of Prosthetic Dentistry, Medical Faculty Heidelberg, Heidelberg University, Im Neuenheimer Feld 400, 69120 Heidelberg, Germany; wolfgang.boemicke@med.uni-heidelberg.de; 6 Private Dental Practice, Am Rain 2, 04178 Leipzig, Germany; ritabensel@zahnarzt-am-rain.de; 7 Institute for Research in International Assistance, Akkon University for Human Sciences, Colditzstraße 34-36, 12099 Berlin, Germany

**Keywords:** oral health-related quality of life, children, child oral impact on daily performances, dental caries, oral hygiene, Tanzania

## Abstract

**Highlights:**

**What are the main findings?**

- Missing teeth, toothbrushing-related symptoms, and high sugar intake were the strongest predictors of impaired oral health-related quality of life among schoolchildren in rural Tanzania.
- Eating was the most frequently affected daily activity, largely driven by tooth exfoliation and dental pain, with 80.5% of children caries-free yet reporting substantial oral health impacts.

**What are the implications of the main findings?**

- School-based oral health programs combining hygiene education, supervised brushing, and dietary counseling are needed in rural, resource-limited settings to reduce the burden of untreated oral conditions.
- Integrating oral health into primary healthcare services and prioritizing early screening may help mitigate the functional and psychosocial consequences of oral disease in underserved pediatric populations.

**Abstract:**

Objectives: Oral health-related quality of life (OHRQoL) reflects the functional and psychosocial impacts of oral conditions on daily life. In low-resource settings such as rural Tanzania, limited access to dental care and preventive services may increase the burden of oral disease. This study assessed the association between clinical oral health conditions and OHRQoL among schoolchildren in rural Tanzania. Methods: A cross-sectional study was conducted among 293 schoolchildren at Igelehezda Primary School, Ilembula, Tanzania. Clinical examinations assessed dental caries using the DMFT index and oral hygiene using the OHI-S index. OHRQoL was measured with the Child Oral Impact on Daily Performances (C-OIDP) questionnaire. Behavioral data included sugar intake, number of daily meals, and toothbrushing-related symptoms. Associations between clinical, behavioral factors and OHRQoL were analyzed using descriptive statistics, bivariate tests, and multiple linear regression (*p* < 0.05). Results: All 293 children completed the study (mean age 12.2 ± 1.2 years; 157 females, 136 males). Mean DMFT was 2.7 ± 4.1, with 80.5% free of untreated caries, and mean OHI-S indicated good oral hygiene (0.4 ± 0.6). Most participants were periodontally healthy (68.3%). Toothache, gum pain, or bleeding during brushing were reported by 26.0–31.6%. Eating was the most affected daily activity (42.7%). Missing teeth, toothbrushing-related symptoms, and consumption of high-sugar sweets were significantly associated with higher C-OIDP scores (*p* < 0.05), while a higher number of daily meals was associated with fewer impacts. Conclusions: Missing teeth, toothbrushing-related symptoms, and high sugar intake were associated with greater impairment in daily life, particularly affecting eating. These findings highlight the need for preventive and educational oral health interventions in rural, resource-limited settings.

## 1. Introduction

Oral health-related quality of life (OHRQoL) in children encompasses four key dimensions: oral function, orofacial pain, orofacial appearance, and psychosocial impact [1]. These dimensions reflect the broader understanding that oral health is not merely the absence of disease but a fundamental component of general health and well-being, influencing physical comfort, emotional stability, and social functioning [2,3].

Globally, dental caries and gingival diseases represent the most prevalent oral diseases and can substantially impair OHRQoL [3,4,5]. In addition, tooth loss—including premature loss of primary teeth and loss of permanent teeth—can substantially affect OHRQoL in children by impairing mastication, speech, and psychosocial well-being [5]. Their impact is particularly profound in children, for whom oral health issues can interfere with essential aspects of development, such as nutrition, communication, school performance, and self-esteem [6]. Establishing effective preventive strategies and educational programs in early childhood is therefore critical to promoting long-term oral health and quality of life [7,8].

Especially in low- and middle-income countries (LMICs), the capacity for oral health is often compromised due to socioeconomic disparities, infrastructural deficiencies, and limited access to health education and services [9]. Consequently, the burden of oral disease in these settings tends to be higher and more persistent, with significant implications for the overall quality of life [1].

Historically, dental caries prevalence in many low- and middle-income countries, including those in East Africa, was comparatively lower than in high-income regions. For example, meta-analytic data from East Africa indicate a pooled caries prevalence of approximately 30–45%, with Tanzania among the lower end of this range [10]. However, since the political and economic liberalization of East Africa in the early 1980s, a marked increase in the consumption of refined sugars has led to a rise in dental caries, also documented in Tanzanian populations [11,12,13]. Despite this trend, the prevalence of caries remains below that observed in high-income countries and may be influenced by several factors such as brushing frequency, parental education and oral care practices [14].

Tanzania, located in East Africa with a population of approximately 68.6 million inhabitants (mid-2024 estimate), faces substantial socioeconomic challenges, with around 40–43% of the population living below the international poverty line [15,16,17]. Due to economic growth in 2020, the country transitioned from low-income to LMIC status. Oral healthcare provision remains limited. Tanzania’s dentist-to-population ratio is approximately 0.1–0.12:10,000, which is significantly below the African average of 0.37–0.44:10,000 and far below the ratio of 7.6–7.9:10,000 found in high-income countries [18,19,20].

The shortage of personnel is exacerbated by limited training opportunities for oral healthcare providers in Tanzania and the fact that dental services are predominantly offered in the public sector with minimal reach into rural areas, particularly in the highland regions [21,22]. The lack of infrastructure, preventive programs, and trained personnel severely restricts access to quality oral healthcare.

Combined with the rising consumption of cariogenic foods and the absence of comprehensive prevention strategies, these conditions contribute to the growing prevalence of oral diseases in Tanzania. Such diseases are likely to impair oral function, negatively affecting children’s OHRQoL [23,24].

OHRQoL is typically measured using validated questionnaires designed to capture the subjective impact of oral conditions on daily functioning. These instruments provide insights into how clinical oral health status affects everyday life, enabling a comprehensive assessment of both clinical and psychosocial outcomes. The Oral Impact on Daily Performances (OIDP) questionnaire, for example, is a confirmed tool for evaluating the frequency, severity, and impact of oral problems on daily life [25,26,27].

The Child Oral Impact on Daily Performances (C-OIDP) is the measurement instrument used in this study. It was developed from the OIDP and first tested on schoolchildren in Thailand by Gherunpong in 2004 [25,28]. The structure of the questionnaire is similar to the OIDP. With this instrument, the impact of oral problems on eight different activities in children’s daily lives can be assessed.

While several studies have investigated oral health status in Tanzanian children, few have combined standardized clinical indices (DMFT, OHI-S, CPI) with a validated child-specific OHRQoL instrument such as the C-OIDP in a rural highland setting. Most existing data originate from urban or peri-urban areas, leaving a significant gap in understanding the oral health burden and its impact on daily functioning among children in remote, resource-limited communities.

This study aimed to assess the relationship between clinical oral health conditions (dental caries, oral hygiene status, tooth loss) and oral health–related quality of life among schoolchildren from 5th to 7th class, in the Ilembula region of Tanzania. Specifically, the study sought to identify which clinical and behavioral factors most strongly predict impacts on daily performances as measured by the C-OIDP-index.

The null hypothesis for this study was that there is no significant association between clinical oral health parameters (DMFT, OHI-S, CPI, missing teeth) or behavioral factors (sugar intake, number of meals per day, symptoms during toothbrushing) and oral health–related quality of life, as measured by the C-OIDP-index.

## 2. Materials and Methods

### 2.1. Participants

A probability sampling method was employed for participant recruitment from the student population of the Igelehezda Primary School. Students were selected based on a comprehensive list of all pupils from 5th to 7th class, ensuring each eligible student had an equal chance of being included in the sample.

A total of 293 school children participated in this cross-sectional study. The sample size was based on the number of children available during the study period. Participants who did not complete more than 20% of the questionnaires or who were missing parts of the clinical examination were excluded. In practice, no participants were excluded based on these criteria, as all 293 children provided sufficiently complete data. No formal a priori sample size calculation or power analysis was performed; this is acknowledged as a limitation of the study. Nevertheless, the sample of 293 participants is comparable to or exceeds sample sizes reported in similar cross-sectional studies of OHRQoL in East African schoolchildren [25,26], and the number of observed events was sufficient to detect statistically significant associations in the regression models.

Data was collected in November 2023. Prior to participation, written informed consent was obtained from all parents/guardians. Approval for the human research was obtained from the National Institute for Medical Research (NIMR), Tanzania (NIMR/HQ/R.8a/vol. IX/3192), and this study was conducted in accordance with the principles of the Declaration of Helsinki.

To participate in this study, participants had to fulfill the following inclusion criteria: they had to be students at the Igelehezda Primary School in 5th to 7th class and have parental consent. Participation in this study occurred during regular school time and was voluntary. Exclusion criteria included students with severe medical conditions that would impede oral examination and those lacking parental consent. Participation was voluntary, and children were free to decline or withdraw at any time.

### 2.2. Study Site

The study took place in the community of Ilembula, which is one of the 21 districts of Wanging’ombe, located in the North of the region Njombe in Tanzania. Ilembula is located in the southwestern highlands of Tanzania and is classified as a rural area due to its average distance of approximately 14 h by car from the country’s economic center, Dar es Salaam. According to the 2022 Tanzanian census, the population of Ilembula is 14,451.

The Igelehezda Primary School was selected using convenience sampling due to its accessibility and representativeness of primary schools in the region. The school serves as an educational institution for children from the surrounding communities and provides a structured environment conducive to conducting the study during regular school hours.

### 2.3. Data Collection

All participants completed two questionnaires and underwent an oral examination according to the WHO guidelines.

The first questionnaire was in hard copy format and was designated as the “Ilembula Data Collection Form—Oral Health” [12]. The questionnaire was based on the guidelines for the design of case-report forms and was designed as a closed structure [29]. It assessed the following items:

1. Personal data, including age, sex, and grade;
2. Socioeconomic information, encompassing general disease, infectious diseases, impaired general wound healing disorders, medication intake, and existing pregnancy (for female participants);
3. Oral hygiene habits, i.e., frequency of brushing, dental care habits, including dental care products;
4. Medical history and dental history;
5. Social and dietary behaviors;
6. Dental status, which was recorded through a clinical examination.

Furthermore, OHRQoL was assessed using the C-OIDP questionnaire [28]. Participants were asked to report whether they had experienced any oral health-related difficulties affecting the following daily performances: eating, speaking, cleaning teeth, sleeping or relaxing, emotional status, smiling, schoolwork, and social contact. For each affected performance, the frequency and severity of the impact were recorded using ordinal response scales. Higher scores indicated a greater negative impact on daily life. Three scoring methods were applied in the present study: simple count score, additive score, and sum score:

1. The simple count score was calculated as the total number of daily performances for which at least one oral health impact was reported. Scores ranged from 0 to 8, with higher values indicating a greater number of affected daily activities.
2. For the additive score, the frequency and severity scores for each impacted performance were multiplied to generate an individual performance score. The additive score was then obtained by summing these performance scores across all eight daily activities. This approach reflects both the extent and intensity of oral health impacts.
3. The sum score was calculated by summing the reported frequency and severity values across all impacted performances without weighting individual activities. This score provides an overall measure of the perceived burden of oral health problems on daily functioning.

Both questionnaires were available in both English and Kiswahili. The Kiswahili version of the C-OIDP has been previously validated for use in Tanzanian populations [25,30]. Kiswahili is the national language of Tanzania and the primary language of instruction at the study school. The questionnaires were administered in a guided, interviewer-assisted format. Additionally, prior to data collection, the questionnaires were pre-tested with the bilingual study administrator to ensure clarity and cultural appropriateness. The bilingual (Kiswahili and English) study support administrator was available during the administration of the questionnaires to answer any queries or other aspects of the study in the participants’ native languages.

### 2.4. Clinical Examination

This study included a single examination of the oral cavity. The clinical examination was conducted by two principal examiners (TB, AH) and additional trained dental examiners, all of whom had received training and had experience in oral health screenings. Prior to data collection, all examiners underwent calibration for the assessment of clinical indices and oral lesions using mounted teeth, slides, and images. Intra- and inter-examiner reliability was assessed: a total of 10 randomly selected subjects were examined twice. Cohen’s kappa values ranged between 0.70 and 0.80 for both intra- and inter-examiner agreement. For the DMFT index specifically, a consensus rate of 94% was achieved, which was considered satisfactory.

The examinations were conducted in the open corridors of the school under daylight or, if not sufficient, with an additional light source. In accordance with the WHO recommendations for oral health screening, the instruments included a dental mirror, dental probe, WHO probe, rubber gloves, and a face mask. All instruments were disposable and were discarded after each examination.

The decayed, missing, and filled teeth (DMFT) indices were employed to document the prevalence of decayed, missing, and filled teeth. Caries was identified in accordance with the WHO criteria at the cavitation level; non-cavitated (initial) lesions were not recorded as carious. Given the age range of participants (11–15 years), the analysis focused on the permanent dentition. Primary teeth were documented during clinical examination but were not included in the DMFT analysis, consistent with WHO recommendations for this age group.

The simplified oral hygiene index (OHI-S) was used to assess oral hygiene and to detect calculus and dental plaque. During the assessment, the presence of Debris (DI-S) and Calculus (CI-S) was evaluated on a scale from 0 to 3 (0 = no debris/calculus visible, 1 = up to 1/3 of the tooth surface covered, 2 = 1/3 to 2/3 covered, 3 = more than 2/3 covered). The following tooth surfaces were evaluated: the buccal surfaces of teeth 16, 11, 26, and 31, and the lingual surfaces of teeth 36 and 46 (FDI notation). In case of missing teeth, the respective distal tooth was assessed.

The OHI-S score was calculated by adding the DI-S and CI-S indices and dividing by the number of assessed areas, resulting in values from 0 to 6. Scores were categorized as good oral hygiene (<1.2), fair oral hygiene (1.2–3.0), and poor oral hygiene (3.1–6.0).

The Angle Classification was used to evaluate bite position.

If present, the following intraoral characteristics were considered: medial diastema, gingival recession, and tooth wear. The degree of wear was determined based on the presence or absence of enamel surface features. The Eichner and Kennedy classifications were used to characterize partial edentulism. The effects of clinically apparent oral habits (thumb sucking, nail biting, bruxism) were recorded when present. During clinical examination, the teeth, oral cavity, and both jaws were examined. The occurrence of intraoral anomalies, including crossbites, tooth position anomalies, dental trauma, recession, gingival hyperplasia, and mucosal diseases, was documented.

### 2.5. Data Analysis

Statistical analyses were performed using Stata/IC 16.1 for Unix (StataCorp, 4905 Lakeway Drive, College Station, TX 77845, USA). Descriptive statistics (means, standard deviations, frequencies) were calculated for all variables. Preliminary bivariate analyses were conducted to screen for associations between demographic, behavioral, clinical, and OHRQoL variables and to identify candidates for multivariable modeling. Multiple linear regression analyses were used to examine predictors of oral health outcomes (DMFT and OHI-S). Variable selection was based on theoretical relevance, previous evidence, and a liberal inclusion criterion of *p* < 0.25 in bivariate analyses. Variables meeting this threshold were entered as candidate predictors in the multivariable models; the final models were built using a combination of theoretical relevance and statistical significance. Standard regression diagnostics were applied to assess assumptions of linearity, normality, homoscedasticity, and multicollinearity (assessed via variance inflation factors [VIF]); no major violations were detected. Standardized beta coefficients (β) and adjusted R^2^ values were reported. Statistical significance was set at *p* < 0.05. For Child-OIDP outcomes (additive score, simple count, sum score), group differences and associations with clinical or behavioral factors were examined using linear regression models. All tests were two-tailed.

## 3. Results

### 3.1. Participant Characteristics

A total of 293 participants were included in the final descriptive analysis (157 females and 136 males aged 11–15 years; mean age 12.2 ± 1.2 years). The age gap was due to school children who were repeating grades or late enrollment into the first grade. Descriptive analyses of oral health-related quality of life included all available cases.

### 3.2. Oral Health Status

#### 3.2.1. Dental Caries Experience (DMFT Index)

The mean DMFT score in the total study population was 2.7 ± 4.1, with a median of 1.0 (IQR: 0–4; range: 0–16), indicating a generally low but heterogeneous caries experience. Mean DMFT values were higher in males compared to females (Mean: males 3.0, females 2.5), and variability was slightly higher among males (SD: 4.5 vs. 3.6) (Table 1).

Regarding individual DMFT components, the mean number of decayed teeth was 0.5 ± 1.2, with 80.5% of children presenting no untreated carious lesions. At least one decayed tooth was observed in 7.5% of the participants. The mean number of missing teeth was 2.3 ± 4.1, while no filled teeth were recorded in the study population, resulting in a mean filled component of 0.0 across all participants (Table 2).

Linear regression analysis identified age as the only factor significantly associated with DMFT scores. Sex, tooth brushing frequency, number of daily meals, sweet consumption, intake of sugared tea, and soda consumption were not significantly associated with DMFT scores (*p* > 0.05 for all) (Table 3).

#### 3.2.2. Community Periodontal Index (CPI)

The median highest CPI score was 0.0 (IQR 0.0–1.0) with a mean of 0.38 ± 0.61. The majority of participants (68.3%, *n* = 200) were periodontally healthy (CPI 0), while 25.3% (*n* = 74) presented with bleeding on probing (CPI 1) and 6.5% (*n* = 19) had calculus (CPI 2). No participants showed signs of periodontal pocketing (CPI 3–4) (Table 4).

Female participants had a higher prevalence of gingival inflammation (CPI ≥ 1: 38.2%) compared to males (26.1%). A trend toward increasing CPI scores with age was observed, with the proportion of healthy periodontia (CPI 0) decreasing from 77.5% at age 11 to 54.8% at age 14 (Table 4).

#### 3.2.3. Oral Hygiene Status (OHI-S)

The mean Oral Hygiene Index–Simplified (OHI-S) score for the total sample was 0.4 ± 0.6, with a median of 0.0 (IQR: 0.0–1.0; range: 0.0–2.0), corresponding to an overall classification of good oral hygiene. Mean OHI-S scores were slightly higher in males (0.4 ± 0.5) compared with females (0.3 ± 0.6) (Table 5).

Regression analysis showed no statistically significant associations between OHI-S scores and age, sex, brushing frequency, number of daily meals, or consumption of sweets, sugared tea, or sodas. Although soda consumption showed a trend towards an inverse association with OHI-S scores (95% CI: −0.19 to 0.00; *p* = 0.086), this relationship did not reach statistical significance (Table 6).

#### 3.2.4. Angle Classification

Angle classification was uniform across the study population, with a mean value of 1.0 ± 0.0 and no observed variability between females and males (Table 5).

### 3.3. Oral Care Practices

Self-reported oral hygiene practices varied among participants. Regarding brushing frequency among respondents, 46.9% reported brushing their teeth once per day, while 53.1% reported brushing twice or more frequently per day (Table 7).

With regard to oral hygiene tools, the majority of participants reported using a toothbrush (87.2%) and toothpaste (53.8%). The use of adjunctive cleaning aids was less common: only 12.1% reported using toothpicks, and 1.7% reported using dental floss. A minority of participants (12.8%) indicated that they did not use a toothbrush (Table 7).

Symptoms experienced during tooth brushing were frequently reported. Toothache while brushing was reported by 31.6% of participants, gum pain by 26.0%, and gingival bleeding by 29.5%. Additionally, 26.2% of the children reported experiencing toothache within the week preceding the survey (Table 7).

### 3.4. Quality of Life Assessment

Descriptive analysis of the C-OIPD questionnaire revealed that toothache (39.6%) and tooth exfoliation (56.0%) were the most commonly experienced oral problems. It was noticeable that the prevalence of oral problems decreased with higher school class within the same oral problem (Table 8).

Eating was the daily performance most frequently affected among the assessed schoolchildren (Table 9). Overall, 42.7% of participants reported experiencing difficulties while eating at least once or twice per month. Tooth exfoliation appeared to be a major contributing factor, as 52.4% of the children reported difficulties associated with exfoliating teeth. Among these, 62.5% indicated that such difficulties occurred almost daily. In addition, nearly half of the children (47.4%) reported eating difficulties related to toothache (Table 10).

Regarding the perception and satisfaction with the oral cavity, it was recognizable that 37.9% of children were dissatisfied with their mouth/teeth, and 37.0% thought that their teeth were in a bad state. Most school children answered to be dissatisfied with the color (40.1%) and appearance (41.3%) of their teeth (Table 11).

### 3.5. C-OIDP Additive, Simple Count and Sum Scores

The Child-Oral Impact on Daily Performances (C-OIDP) questionnaire revealed that a substantial proportion of children experienced oral impacts affecting their daily lives. The most frequently impacted activities were eating, cleaning teeth, and emotional state, while impacts on speaking, sleeping, and social contact were less common.

Child-OIDP Additive Score

Significant associations were observed between several clinical and behavioral variables and the additive C-OIDP score (Table 12).

- Missing teeth showed a strong positive association with higher impairment (*p* < 0.001).
- Meals per day were negatively associated with impact (*p* = 0.023), indicating that children consuming more meals reported fewer daily limitations.
- Symptoms during toothbrushing demonstrated the most consistent and strongest associations:
  - Toothache while brushing (*p* < 0.001);
  - Gum pain while brushing (*p* = 0.006);
  - Gum bleeding while brushing (*p* < 0.001).

**Table 12:** Child-OIDP Additive Score—Metric Characteristics.

	*n*	Coeff. [95%-CI]	*p*-Value
Age	186	−0.30 [−1.06 to 0.45]	0.433
Decayed teeth	186	0.11 [−0.48 to 0.69]	0.722
Missing teeth	186	0.52 [0.28 to 0.76]	<0.001
OHI-Score	186	1.26 [−0.18 to 2.70]	0.086
Brushing teeth per day	167	−0.10 [−1.25 to 1.05]	0.860
Meals per day	184	−1.75 [−3.60 to 0.10]	0.063
Sweets per day	179	−0.06 [−0.83 to 0.71]	0.875
Sugared tea per day	179	−0.29 [−1.00 to 0.43]	0.425
Sodas per day	178	0.38 [−0.83 to 1.59]	0.532

Child-OIDP Simple Count Score

The simple count score (number of daily activities affected) revealed similar patterns (Table 13):

- Meals per day again showed a protective effect (*p* = 0.015).
- Toothbrushing-related symptoms were strongly associated with an increasing number of affected activities:
  - Toothache (*p* < 0.001);
  - Gum pain (*p* = 0.008);
  - Gum bleeding (*p* < 0.001).
- C-OIDP simple count depends statistically significant of the number of missing teeth (*p* < 0.001).

**Table 13:** Child-OIDP Simple Count—Metric Characteristics.

	*n*	Coeff. [95%-CI]	*p*-Value
Age	186	−0.34 [−0.81 to 0.12]	0.148
Decayed teeth	186	0.05 [−0.30 to 0.41]	0.764
Missing teeth	186	0.31 [0.21 to 0.41]	<0.001
OHI-Score	186	0.74 [−0.02 to 1.51]	0.057
Brushing teeth per day	167	−0.13 [−0.72 to 0.47]	0.675
Meals per day	184	−1.11 [−1.99 to −0.22]	0.015
Sweets per day	179	0.08 [−0.42 to 0.58]	0.752
Sugared tea per day	179	−0.08 [−0.58 to 0.42]	0.758
Sodas per day	178	0.37 [−0.33 to 1.07]	0.300

Child-OIDP Sum Score (Impact × Frequency)

The C-OIDP sum score, which reflects both the severity and frequency of impacts, demonstrated additional significant associations (Table 14):

- Sweets per day showed a positive and significant association with the sum score (*p* = 0.042), indicating a worsening of impact with higher sugar intake.
- Missing teeth, toothache, gum pain, and gum bleeding also showed consistently strong associations with higher sum scores (all *p* < 0.01).

**Table 14:** Child-OIDP Sum Score—Metric Characteristics.

	*n*	Coeff. [95%-CI]	*p*-Value
Age	164	0.01 [−0.32 to 0.34]	0.948
Decayed teeth	164	0.19 [−0.19 to 0.58]	0.317
Missing teeth	164	0.09 [−0.01 to 0.20]	0.076
OHI-Score	164	0.20 [−0.60 to 1.01]	0.620
Brushing teeth per day	144	0.54 [−0.04 to 1.12]	0.066
Meals per day	161	−0.49 [−1.24 to 0.27]	0.208
Sweets per day	156	0.44 [0.02 to 0.86]	0.042
Sugared tea per day	160	0.22 [−0.35 to 0.79]	0.451
Sodas per day	158	0.37 [−0.20 to 0.94]	0.206

## 4. Discussion

This study revealed clear associations between clinical oral conditions and OHRQoL among schoolchildren in rural Tanzania. The most consistent predictor of reduced OHRQoL across all Child-OIDP indices was Missing teeth, which showed one of the strongest associations with higher OIDP scores (*p* < 0.001), indicating substantial functional and psychosocial impact.

Notably, the relatively low untreated caries prevalence (80.5% caries-free) coexists with significant OHRQoL impairment. This apparent discrepancy can be explained by the fact that OHRQoL instruments such as the C-OIDP capture a broader spectrum of oral health experiences beyond untreated caries alone. In this study population, dental pain was reported by 39.6% of children, active tooth exfoliation by 56.0%, and gingival symptoms including bleeding and swelling were common. These conditions, together with the psychological impact of missing teeth and dissatisfaction with dental appearance, are likely to contribute substantially to impaired daily functioning, even in the absence of high caries prevalence. This underscores the importance of assessing OHRQoL alongside clinical indices, as clinical parameters alone may underestimate the perceived burden of oral conditions.

An inverse association between age and DMFT was identified, with lower DMFT values observed among older participants. While dental caries is commonly regarded as cumulative, this finding may be related to the specific age structure of the study population. Within the relatively narrow age range of school-aged children, DMFT may not increase uniformly with age, as younger children may present with higher scores due to the vulnerability of newly erupted permanent teeth, whereas older children are more likely to have undergone extraction of decayed teeth or exfoliation of primary teeth, thereby reducing the D component. The complete absence of filled teeth (F = 0) across the entire study population is a striking finding that directly reflects the extremely limited access to restorative dental care in rural Tanzania, where the dentist-to-population ratio is approximately 0.1:10,000 and dental services are virtually absent in highland rural areas [17,20,21]. In such settings, extraction remains the predominant treatment modality, which further contributes to the high M component observed. Comparable research in pediatric populations suggests that the relationship between age and DMFT is complex and may not be strictly linear across narrow age ranges [31,32,33].

Furthermore, symptoms during toothbrushing, including toothache, gum pain, and gingival bleeding, were significantly linked to greater daily life impairment (*p* < 0.01 to *p* < 0.001). High sugar foods, which significantly increased the OIDP sum score (*p* = 0.042), point to a relationship between dietary habits and perceived oral health impact. Meals per day, which acted as a protective factor, showed a negative association with OIDP scores (*p* ≈ 0.015–0.023).

The observed association between a higher number of daily meals and better oral health-related quality of life should be interpreted with caution. Rather than reflecting a direct protective effect, meal frequency may act as a proxy for structured daily routines, caregiving patterns, or broader socioeconomic factors that positively influence perceived well-being [34,35].

These overall findings illustrate that both clinical findings (tooth loss, gum symptoms) and behavioral factors (dietary habits, brushing-related symptoms) are associated with children’s perceived oral health and daily functioning.

The results found in this cross-sectional study align with previous studies conducted in LMIC, which likewise report that oral diseases have a negative effect on children’s daily activities and psychosocial well-being. A scoping review conducted by Kafayat Aminu et al. evaluated the oral health status in relation to OHRQoL across the East African community. They found that students from urban areas of Tanzania had problems mainly while eating, social contact and smiling, like the results of this study. Furthermore, a prevalence of 23% in underaged children was found regarding tooth pain, which is slightly lower compared to the results found here (39.6%). This difference may be attributable to several factors, including the rural setting of the present study, where access to dental care is more restricted, potentially leading to a higher burden of untreated symptomatic conditions, as well as differences in dietary habits and oral hygiene practices between rural and urban populations. Nevertheless, the clearest agreement of the studies was the association between high sugar intake and higher OIDP-scores, indicating a significant negative impact of high sugar intake on daily performances. Contrary results were found regarding perception of teeth/mouth. Around 90% of children were satisfied and happy with their teeth’s appearance, which contradicts the results found in this study. However, this discrepancy may be explained by differences in the age range of participants, the specific wording and response scales of satisfaction-related questions, as well as culturally shaped expectations and norms regarding dental aesthetics, which may vary considerably between study settings [36].

Furthermore, Malele-Kolisa et al. also found a strong association between any oral condition and a poorer OHRQoL in their systematic review [37]. It was noticeable that poor oral conditions had a higher influence on OHRQoL than emotional and social well-being. It was found that caries experience did have the greatest impact on OHRQoL among children. In the present study, caries prevalence was relatively low, which may be explained by the age distribution of the participants, as many children were transitioning from primary to newly erupted permanent dentition, which may not yet show advanced carious lesions [37].

On the other hand, the cross-sectional study conducted by Gherunpong et al. also found a low caries prevalence with a medium DMFT score of 1, exactly as found in this study [25]. The study took place in Thailand and used the same C-OIDP index. Therefore, the results can be compared satisfactorily. They also saw, that the ORHQoL was a particularly diminished by eating difficulties and discomfort while smiling. These results align with the ones found in this study [25].

Contrastingly to the results found in LMIC, studies in high-income countries often report higher prevalence of restored teeth (filled component) and lower functional impairment, suggesting that differences in dental care systems, access, and preventive strategies play an important role in shaping OHRQoL outcomes towards positive [38].

Several contextual factors likely explain the findings, such as limited access to dental care in rural Tanzania (very low dentist-to-population ratio), which increases the likelihood that oral problems remain untreated, leading to pain, tooth loss, and functional limitations. Furthermore, the lack of preventive programs, including fluoride availability and school-based oral health education, may contribute to persistent gingival problems and early tooth decay. Dietary transitions, including increased consumption of sugary foods and beverages in rural East Africa, may explain the link between sweet intake and higher OIDP scores [12,38,39].

Public health implications for example include the need to establish school-based oral health programs, including hygiene education, supervised brushing, and early detection of dental problems. Prioritizing dietary education aimed at reducing sugar intake, would be probably given its measurable impact on OHRQoL. Finally, integrating oral health more closely into primary healthcare services, particularly in rural districts with extremely limited dental resources.

Overall, the findings indicate a need for preventive and educational interventions tailored to rural Tanzanian schoolchildren. Aligning with the results from the cross-sectional study by authors Masumo et al., it can be suggested that children may benefit from early screening and prevention programs [40].

A major strength of this study is the use of a validated OHRQoL instrument (Child-OIDP), enabling reliable assessment and comparison with international literature. The relatively large sample size enhances the robustness of the findings within the study context, and the combination of subjective OHRQoL measures with objective clinical assessments provides a comprehensive view of oral health impacts.

Nevertheless, some limitations should be considered when interpreting the results. The cross-sectional design prevents establishing causal relationships between clinical status and OHRQoL. Furthermore, some data was self-reported, which may introduce the possibility of bias as well. In addition, the use of paper-based questionnaires may have introduced ambiguity in cases of incomplete or unclear responses. Importantly, all participants were drawn from a single school in the Ilembula region, which was selected by convenience due to an existing institutional collaboration. This limits the generalizability of the findings beyond this specific setting, and the results should not be extrapolated to the broader Tanzanian schoolchild population without caution. No formal a priori sample size calculation was performed, which may have limited the statistical power to detect smaller effect sizes. The use of a liberal screening threshold (*p* < 0.25) for regression variable selection, while common in exploratory analyses, may have introduced variables with limited predictive value into the models. Finally, the omission of primary dentition from the DMFT analysis, while consistent with WHO guidelines for this age group, may have underestimated the total caries burden, particularly among younger participants in the transitional dentition phase.

Future research should prioritize longitudinal designs to examine how oral health status and OHRQoL evolve over time and to assess the effectiveness of preventive interventions such as supervised brushing, fluoride application, and dietary education. Incorporating qualitative methods, including interviews with children and caregivers, may provide deeper insight into psychosocial dimensions of oral health. Comparative studies between rural and urban populations would further enhance understanding of geographic disparities in OHRQoL within LMIC settings.

## 5. Conclusions

In this cross-sectional study of schoolchildren at a single primary school in rural Tanzania, oral health conditions were associated with a substantial burden on oral health-related quality of life. Missing teeth and symptoms experienced during toothbrushing—particularly toothache, gum pain, and gingival bleeding—showed the most consistent associations with impaired daily functioning across all Child-OIDP indices. Eating was the most frequently affected daily activity, largely associated with tooth exfoliation and dental pain. Higher sweet consumption was associated with more severe and frequent oral health impacts. While these findings cannot be generalized beyond the study population, they suggest a considerable burden of untreated oral conditions in this setting and point to the potential value of preventive, educational, and early intervention strategies tailored to resource-limited rural communities.

## Figures and Tables

**Table 1:** Description of DMFT.

		*n*	Mean	SD	Median	IQR	Min–Max
Decayed teeth	Female	157	0.5	1.2	0.0	0–0	0–8
	Male	136	0.4	1.1	0.0	0–0	0–7
	Total	293	0.5	1.2	0.0	0–0	0–8
Missing teeth	Female	157	2.0	3.7	0.0	0–4	0–16
	Male	136	2.6	4.4	0.0	0–4	0–16
	Total	293	2.3	4.1	0.0	0–4	0–16
Filled teeth	Female	157	0.0	0.0	0.0	0–0	0–0
	Male	136	0.0	0.0	0.0	0–0	0–0
	Total	293	0.0	0.0	0.0	0–0	0–0
DMFT	Female	157	2.5	3.6	1.0	0–4	0–16
	Male	136	3.0	4.5	1.0	0–4	0–16
	Total	293	2.7	4.1	1.0	0–4	0–16

**Table 2:** D, M, F—Number of Teeth.

		Female	Male	Total
Decayed teeth	0	123 (78.3%)	113 (83.1%)	236 (80.5%)
	1	13 (8.3%)	9 (6.6%)	22 (7.5%)
	2	8 (5.1%)	6 (4.4%)	14 (4.8%)
	3	5 (3.2%)	2 (1.5%)	7 (2.4%)
	4	7 (4.5%)	4 (2.9%)	11 (3.8%)
	5	0 (0%)	1 (0.7%)	1 (0.3%)
	7	0 (0%)	1 (0.7%)	1 (0.3%)
	8	1 (0.6%)	0 (0%)	1 (0.3%)
Missing teeth	0	103 (65.6%)	81 (59.6%)	184 (62.8%)
	1	9 (5.7%)	9 (6.6%)	18 (6.1%)
	2	4 (2.5%)	6 (4.4%)	10 (3.4%)
	3	1 (0.6%)	0 (0%)	1 (0.3%)
	4	16 (10.2%)	17 (12.5%)	33 (11.3%)
	5	3 (1.9%)	2 (1.5%)	5 (1.7%)
	7	1 (0.6%)	0 (0%)	1 (0.3%)
	8	10 (6.4%)	6 (4.4%)	16 (5.5%)
	9	1 (0.6%)	1 (0.7%)	2 (0.7%)
	10	0 (0%)	2 (1.5%)	2 (0.7%)
	12	6 (3.8%)	4 (2.9%)	10 (3.4%)
	13	0 (0%)	1 (0.7%)	1 (0.3%)
	16	3 (1.9%)	7 (5.1%)	10 (3.4%)
Filled teeth	0	157 (100%)	136 (100%)	293 (100%)

**Table 3:** DMFT—Linear Regression.

	Coeff. (95%-CI)	*p*-Value
Age (years)	−1.41 (−1.95 to −0.88)	<0.001
Male vs. female	1.07 (−0.01 to 2.15)	0.052
Brushing teeth per day	0.21 (−0.31 to 0.73)	0.421
Meals per day	−0.71 (−1.70 to 0.28)	0.160
Sweets per day	−0.35 (−0.82 to 0.11)	0.138
Sugared tea per day	0.28 (−0.27 to 0.83)	0.311
Sodas per day	0.65 (−0.19 to 1.48)	0.129

**Table 4:** CPI.

CPI	Frequency	Percentage	Valid Percentages	Cumulative Percentages
Good = 0	200	68.3	68.3	68.3
Fair = 1	74	25.3	25.3	93.5
Poor = 2	19	6.5	6.5	100
Total	293	100	100	

**Table 5:** Description of Angle Classification and OHIS-Score.

		*n*	Mean	SD	Median	IQR	Min–Max
Angle classification	Female	157	1.0	0.0	1.0	1.0–1.0	1.0–1.0
	Male	136	1.0	0.0	1.0	1.0–1.0	1.0–1.0
	Total	293	1.0	0.0	1.0	1.0–1.0	1.0–1.0
OHI-Score	Female	157	0.3	0.6	0.0	0.0–0.7	0.0–2.0
	Male	136	0.4	0.6	0.0	0.0–1.0	0.0–2.0
	Total	293	0.4	0.6	0.0	0.0–1.0	0.0–2.0

**Table 6:** OHI-Score—Linear Regression.

	Coeff. (95%-CI)	*p*-Value
Age (years)	0.04 (−0.03 to 0.10)	0.633
Male vs. female	0.15 (−0.00 to 0.31)	0.200
Brushing teeth per day	−0.06 (−0.13 to 0.01)	0.838
Meals per day	−0.02 (−0.16 to 0.12)	0.501
Sweets per day	0.07 (−0.02 to 0.16)	0.105
Sugared tea per day	0.12 (0.02 to 0.22)	0.188
Sodas per day	−0.10 (−0.19 to −0.00)	0.086

**Table 7:** Participant Characteristics: Medical History, Oral Hygiene Practices, Brushing-Related Symptoms, and Dental Treatment History by Sex.

		Female	Male	Total
Gender	Female	-	-	157 (53.6%)
	Male	-	-	136 (46.4%)
Medical History				
Known diseases	No	129 (82.2%)	115 (84.6%)	244 (83.3%)
	Yes	28 (17.8%)	21 (15.4%)	49 (16.7%)
Wound healing	No	143 (91.7%)	133 (97.8%)	276 (94.5%)
	Yes	13 (8.3%)	3 (2.2%)	16 (5.5%)
Infectious diseases	No	151 (96.2%)	127 (93.4%)	278 (94.9%)
	Yes	6 (3.8%)	9 (6.6%)	15 (5.1%)
Medication	No	106 (67.9%)	105 (77.2%)	211 (72.3%)
	Yes	50 (32.1%)	31 (22.8%)	81 (27.7%)
Oral Hygiene Practices				
Brushing teeth per day	1	62 (45.6%)	59 (48.4%)	121 (46.9%)
	2	46 (33.8%)	41 (33.6%)	87 (33.7%)
	≥3	28 (20.6%)	22 (18.0%)	50 (19.4%)
Toothbrush	No	14 (9.1%)	23 (16.9%)	37 (12.8%)
	Yes	140 (90.9%)	113 (83.1%)	253 (87.2%)
Tooth paste	No	65 (42.2%)	69 (50.7%)	134 (46.2%)
	Yes	89 (57.8%)	67 (49.3%)	156 (53.8%)
Dental floss	No	152 (98.7%)	133 (97.8%)	285 (98.3%)
	Yes	2 (1.3%)	3 (2.2%)	5 (1.7%)
Toothpick	No	138 (89.6%)	117 (86.0%)	255 (87.9%)
	Yes	16 (10.4%)	19 (14.0%)	35 (12.1%)
Brushing-Related Symptoms				
Toothache while brushing	No	96 (61.5%)	103 (76.3%)	199 (68.4%)
	Yes	60 (38.5%)	32 (23.7%)	92 (31.6%)
Gums hurt while brushing	No	115 (73.7%)	101 (74.3%)	216 (74.0%)
	Yes	41 (26.3%)	35 (25.7%)	76 (26.0%)
Gums bleed while brushing	No	110 (70.5%)	96 (70.6%)	206 (70.5%)
	Yes	46 (29.5%)	40 (29.4%)	86 (29.5%)
Toothache last week	No	102 (72.9%)	87 (75.0%)	189 (73.8%)
	Yes	38 (27.1%)	29 (25.0%)	67 (26.2%)
Dental Treatment History				
Dental Status without wisdom teeth	Full dentition	104 (66.2%)	81 (59.6%)	185 (63.1%)
	Partially edentulous	53 (33.8%)	55 (40.4%)	108 (36.9%)
Dental treatment in the past 2 years	No	77 (49.4%)	73 (54.5%)	150 (51.7%)
	Yes	79 (50.6%)	61 (45.5%)	140 (48.3%)
Fillings in the past 2 years	No	150 (96.2%)	129 (97.0%)	279 (96.5%)
	Yes	6 (3.8%)	4 (3.0%)	10 (3.5%)
Extraction of a tooth in the past 2 years	No	94 (60.3%)	86 (64.7%)	180 (62.3%)
	Yes	62 (39.7%)	47 (35.3%)	109 (37.7%)
Check up in the past 2 years	No	140 (89.7%)	123 (92.5%)	263 (91.0%)
	Yes	16 (10.3%)	10 (7.5%)	26 (9.0%)

**Table 8:** Self-reported Discomfort in the last 3 Months.

		Total
Toothache	No	172 (60.4%)
*n* = 285	Yes	113 (39.6%)
Sensitive teeth	No	219 (78.5%)
*n* = 279	Yes	60 (21.5%)
Tooth exfoliation	No	125 (44.0%)
*n* = 284	Yes	159 (56.0%)
Problems with the positioning of the teeth	No	250 (87.1%)
*n* = 287	Yes	37 (12.9%)
Ulcer in the mouth	No	200 (70.7%)
*n* = 283	Yes	83 (29.3%)
Bleeding in the mouth	No	218 (77.0%)
*n* = 283	Yes	65 (23.0%)
Swollen gums	No	193 (67.5%)
*n* = 286	Yes	93 (32.5%)
Bad breath	No	198 (69.5%)
*n* = 285	Yes	87 (30.5%)
Problems with the colour of the teeth	No	203 (71.7%)
*n* = 283	Yes	80 (28.3%)
Problems with spaces for the teeth	No	232 (82.0%)
*n* = 283	Yes	51 (18.0%)

**Table 9:** Self-Reported Difficulties Experienced in the last 3 Months.

Difficulty with		Total
Eating	Never	98 (38.4%)
*n* = 255	Once/twice a month	109 (42.7%)
	Once/twice a week	40 (15.7%)
	Everyday/nearly everyday	8 (3.1%)
Speaking	Never	143 (57.0%)
*n* = 251	Once/twice a month	71 (28.3%)
	Once/twice a week	25 (10.0%)
	Everyday/nearly everyday	12 (4.8%)
Cleaning	Never	107 (43.9%)
*n* = 244	Once/twice a month	81 (33.2%)
	Once/twice a week	37 (15.2%)
	Everyday/nearly everyday	19 (7.8%)
Sleeping	Never	138 (54.5%)
*n* = 253	Once/twice a month	70 (27.7%)
	Once/twice a week	35 (13.8%)
	Everyday/nearly everyday	10 (4.0%)
Smiling	Never	144 (57.6%)
*n* = 250	Once/twice a month	63 (25.2%)
	Once/twice a week	32 (12.8%)
	Everyday/nearly everyday	11 (4.4%)
Emotion	Never	151 (61.4%)
*n* = 246	Once/twice a month	57 (23.2%)
	Once/twice a week	29 (11.8%)
	Everyday/nearly everyday	9 (3.7%)
School/Work	Never	151 (63.2%)
*n* = 239	Once/twice a month	56 (23.4%)
	Once/twice a week	23 (9.6%)
	Everyday/nearly everyday	9 (3.8%)
Social contact	Never	146 (59.6%)
*n* = 245	Once/twice a month	57 (23.3%)
	Once/twice a week	30 (12.2%)
	Everyday/nearly everyday	12 (4.9%)

**Table 10:** Self-Reported Difficulties with Oral Cavity/Teeth while Eating During the Past 3 Months.

		Never	Once/Twice a Month	Once/Twice a Week	(Nearly) Everyday	Total
Toothache	No	73 (75.3%)	41 (39.0%)	16 (41.0%)	1 (12.5%)	131 (52.6%)
*n* = 249	Yes	24 (24.7%)	64 (61.0%)	23 (59.0%)	7 (87.5%)	118 (47.4%)
Sensitive teeth	No	86 (90.5%)	80 (78.4%)	29 (76.3%)	7 (87.5%)	202 (83.1%)
*n* = 243	Yes	9 (9.5%)	22 (21.6%)	9 (23.7%)	1 (12.5%)	41 (16.9%)
Tooth exfoliation	No	60 (61.9%)	33 (32.4%)	21 (53.8%)	3 (37.5%)	117 (47.6%)
*n* = 246	Yes	37 (38.1%)	69 (67.6%)	18 (46.2%)	5 (62.5%)	129 (52.4%)
Problems with the position of your teeth	No	88 (90.7%)	91 (85.8%)	33 (84.6%)	7 (87.5%)	219 (87.6%)
*n* = 250	Yes	9 (9.3%)	15 (14.2%)	6 (15.4%)	1 (12.5%)	31 (12.4%)
Ulcer in the mouth	No	82 (85.4%)	77 (74.8%)	24 (64.9%)	7 (87.5%)	190 (77.9%)
*n* = 244	Yes	14 (14.6%)	26 (25.2%)	13 (35.1%)	1 (12.5%)	54 (22.1%)
Bleeding in the mouth	No	87 (89.7%)	71 (67.0%)	27 (71.1%)	5 (62.5%)	190 (76.3%)
*n* = 249	Yes	10 (10.3%)	35 (33.0%)	11 (28.9%)	3 (37.5%)	59 (23.7%)
Swollen gums	No	82 (88.2%)	75 (71.4%)	22 (56.4%)	5 (62.5%)	184 (75.1%)
*n* = 245	Yes	11 (11.8%)	30 (28.6%)	17 (43.6%)	3 (37.5%)	61 (24.9%)
Bad breath	No	83 (84.7%)	62 (61.4%)	22 (56.4%)	8 (100%)	175 (71.1%)
*n* = 246	Yes	15 (15.3%)	39 (38.6%)	17 (43.6%)	0 (0%)	71 (28.9%)
Problems with the colour of your teeth	No	84 (87.5%)	69 (67.0%)	25 (65.8%)	4 (50.0%)	182 (74.3%)
*n* = 245	Yes	12 (12.5%)	34 (33.0%)	13 (34.2%)	4 (50.0%)	63 (25.7%)
Problems with spaces between your teeth	No	78 (82.1%)	86 (81.9%)	30 (83.3%)	6 (75.0%)	200 (82.0%)
*n* = 244	Yes	17 (17.9%)	19 (18.1%)	6 (16.7%)	2 (25.0%)	44 (18.0%)

**Table 11:** Satisfaction and Perception of the Oral Cavity/Teeth.

		Class 5	Class 6	Class 7	Total
What do you think about	Very good	7 (29.2%)	11 (32.4%)	33 (26.2%)	51 (27.7%)
the state of your teeth?	Good	3 (12.5%)	10 (29.4%)	46 (36.5%)	59 (32.1%)
	Bad	13 (54.2%)	13 (38.2%)	42 (33.3%)	68 (37.0%)
*n* = 184	Very bad	1 (4.2%)	0 (0%)	5 (4.0%)	6 (3.3%)
Are you satisfied or dissatisfied	Very satisfied	7 (29.2%)	10 (31.3%)	28 (22.2%)	45 (24.7%)
with your mouth/teeth?	Satisfied	5 (20.8%)	6 (18.8%)	37 (29.4%)	48 (26.4%)
	Dissatisfied	10 (41.7%)	9 (28.1%)	50 (39.7%)	69 (37.9%)
*n* = 182	Very dissatisfied	2 (8.3%)	7 (21.9%)	11 (8.7%)	20 (11.0%)
How satisfied or dissatisfied are you	Very satisfied	6 (28.6%)	8 (27.6%)	39 (32.2%)	53 (31.0%)
with the position of your teeth?	Satisfied	3 (14.3%)	11 (37.9%)	33 (27.3%)	47 (27.5%)
	Dissatisfied	11 (52.4%)	6 (20.7%)	39 (32.2%)	56 (32.7%)
*n* = 171	Very dissatisfied	1 (4.8%)	4 (13.8%)	10 (8.3%)	15 (8.8%)
How satisfied or dissatisfied are you	Very satisfied	4 (16.7%)	6 (18.8%)	31 (25.2%)	41 (22.9%)
with the appearance of your teeth?	Satisfied	4 (16.7%)	11 (34.4%)	37 (30.1%)	52 (29.1%)
	Dissatisfied	12 (50.0%)	10 (31.3%)	52 (42.3%)	74 (41.3%)
*n* = 179	Very dissatisfied	4 (16.7%)	5 (15.6%)	3 (2.4%)	12 (6.7%)
How satisfied or dissatisfied are you	Very satisfied	4 (16.7%)	12 (36.4%)	39 (31.2%)	55 (30.2%)
with the colour of your teeth?	Satisfied	2 (8.3%)	8 (24.2%)	26 (20.8%)	36 (19.8%)
	Dissatisfied	14 (58.3%)	7 (21.2%)	52 (41.6%)	73 (40.1%)
*n* = 182	Very dissatisfied	4 (16.7%)	6 (18.2%)	8 (6.4%)	18 (9.9%)
What do you think about the state	Very good	8 (33.3%)	9 (27.3%)	36 (28.8%)	53 (29.1%)
of your general health?	Good	6 (25.0%)	9 (27.3%)	45 (36.0%)	60 (33.0%)
	Bad	8 (33.3%)	11 (33.3%)	41 (32.8%)	60 (33.0%)
*n* = 182	Very bad	2 (8.3%)	4 (12.1%)	3 (2.4%)	9 (4.9%)

## Data Availability

The data used to support the findings of this study may be released upon an application to the Department of Reconstructive Dentistry and Gerodontology, School of Dental Medicine, University of Bern, which can be contacted through Kyra Michels, Department of Reconstructive Dentistry and Gerodontology, School of Dental Medicine, University of Bern, 3010 Bern, Switzerland.
